# Microbial Tryptophan Metabolites Ameliorate Ovariectomy‐Induced Bone Loss by Repairing Intestinal AhR‐Mediated Gut‐Bone Signaling Pathway

**DOI:** 10.1002/advs.202404545

**Published:** 2024-07-23

**Authors:** Chuan Chen, Zheng Cao, Hehua Lei, Cui Zhang, Mengjing Wu, Shaohua Huang, Xinzhi Li, Denghui Xie, Maili Liu, Limin Zhang, Gang Chen

**Affiliations:** ^1^ State Key Laboratory of Magnetic Resonance and Imaging National Centre for Magnetic Resonance in Wuhan Innovation Academy of Precision Measurement Science and Technology CAS Wuhan 430071 China; ^2^ University of Chinese Academy of Sciences Beijing 100049 China; ^3^ Institute of Drug Discovery and Technology Ningbo University Ningbo 315211 China; ^4^ School of Pharmacy and State Key Laboratory of Quality Research in Chinese Medicine Macau University of Science and Technology Macau 999078 China; ^5^ Department of Joint Surgery Center for Orthopaedic Surgery The Third Affiliated Hospital of Southern Medical University Guangzhou 510515 China; ^6^ Department of Geriatrics Hubei Provincial Hospital of Traditional Chinese Medicine (Affiliated Hospital of Hubei University of Chinese Medicine) Wuhan 430060 China

**Keywords:** aryl hydrocarbon receptor (AhR), gut microbiota, gut‐bone axis, IL‐10, tryptophan (Trp) metabolites, Wnt/β‐catenin signaling

## Abstract

Microbial tryptophan (Trp) metabolites acting as aryl hydrocarbon receptor (AhR) ligands are shown to effectively improve metabolic diseases via regulating microbial community. However, the underlying mechanisms by which Trp metabolites ameliorate bone loss via gut‐bone crosstalk are largely unknown. In this study, supplementation with Trp metabolites, indole acetic acid (IAA), and indole‐3‐propionic acid (IPA), markedly ameliorate bone loss by repairing intestinal barrier integrity in ovariectomy (OVX)‐induced postmenopausal osteoporosis mice in an AhR‐dependent manner. Mechanistically, intestinal AhR activation by Trp metabolites, especially IAA, effectively repairs intestinal barrier function by stimulating Wnt/β‐catenin signaling pathway. Consequently, enhanced M2 macrophage by supplementation with IAA and IPA secrete large amount of IL‐10 that expands from intestinal lamina propria to bone marrow, thereby simultaneously promoting osteoblastogenesis and inhibiting osteoclastogenesis in vivo and in vitro. Interestingly, supplementation with Trp metabolites exhibit negligible ameliorative effects on both gut homeostasis and bone loss of OVX mice with intestinal AhR knockout (Villin^Cre^Ahr^fl/fl^). These findings suggest that microbial Trp metabolites may be potential therapeutic candidates against osteoporosis via regulating AhR‐mediated gut‐bone axis.

## Graphical abstract

Chen et al. identify that supplementation with microbiota‐derived metabolites, indole acetic acid (IAA) and indole‐3‐propionic acid (IPA), can effectively ameliorate bone loss via repairing AhR‐mediated gut‐bone signalling. Mechanistically, activation of AhR binds to the promotor region of β‐catenin gene leading to expansion of interleukin‐10 (IL‐10) from intestinal lamina propria into bone marrow, thereby regulating osteoblast and osteoclast differentiation in vivo and in vitro.

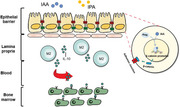

## 1 Introduction

Osteoporosis is a common aging‐related metabolic disease characterized by bone loss and structural deterioration ultimately leading to substantial morbidity and mortality in humans.^[^ ^1^, ^2^, ^3^ ^]^ It is a complicated skeletal degenerative process attributing to many factors such as obesity, aging, and aging‐related diseases.^[^ ^4^, ^5^ ^]^ Typically, postmenopausal osteoporosis in women aged above 50 is mainly due to the decline of ovarian function and estrogen deficiency with high incidence of osteoporotic bone loss and fractures.^[^ ^6^, ^7^ ^]^ At the cellular level, the delicate balance between osteoblasts formation and osteoclasts resorption maintains bone metabolic homeostasis.^[^ ^8^, ^9^, ^10^ ^]^ Estrogen deficiency triggers imbalance between osteoblasts and osteoclasts accompanied by limited osteoclast apoptosis resulting in bone resorption and bone loss,^[^ ^11^ ^]^ which highly impacts patient's life quality. Nowadays, clinical drugs for osteoporosis are bisphosphonates including alendronate, risedronate, zoledronate, and ibandronate that can effectively reduce the risk of nonspine fractures. Unfortunately, orally taken of bisphosphonates may increase risk of over suppressing bone turnover after long‐term taking bisphosphonates. Novel intervention strategies for osteoporosis are therefore urgently needed.

Increasing evidence suggests that many metabolic diseases such as aging, obesity, diabetes, and osteoporosis are closely attributed to intestinal microecology that involves intestinal epithelium, the gut microbiota, and its metabolites.^[^ ^12^, ^13^ ^]^ The intestinal epithelium constitutes a single‐layer barrier that separates the mucosal immune system from trillions of commensal bacteria.^[^ ^14^ ^]^ It is mainly responsible for absorbing nutrients and preventing harmful substances from the gut lumen into lamina propria, thereby maintaining intestinal homeostasis.^[^ ^15^ ^]^ Several types of epithelial cells between the gut lumen and mucosal tissue are very closely interlinked with proteins of adhesive and tight junctions (AJs and TJs) forming the primary barrier and maintaining intestinal barrier integrity.^[^ ^16^ ^]^ Notably, the lamina propria located beneath intestinal epithelium is a habitat for numerous immune cells including lymphocytes, macrophages, and T helper cells (Th1 and Th17) forming intestinal immune barrier.^[^ ^17^, ^18^ ^]^ Intestinal epithelial leakiness due to the increased gut permeability results in activation of host immune response, leading to inflammation and related chronic diseases.^[^ ^16^, ^19^, ^20^ ^]^ Previous studies showed that estrogen deficiency‐induced bone loss was relevant to Th17 cells expansion from the lamina propria to bone marrow, resulting in elevated production of IL‐17 and osteoporosis.^[^ ^21^ ^]^

It is well documented that the aryl hydrocarbon receptor (AhR), a ligand‐dependent transcription factor, plays a pivotal role in mediating host immune response and intestinal homeostasis.^[^ ^22^ ^]^ Nowadays, many physiological AhR ligands such as dietary components and microbial metabolites have been identified to exhibit beneficial functions in different metabolic diseases via activation of AhR for maintenance of intestinal homeostasis.^[^ ^22^ ^]^ Of particular note is that microbial tryptophan (Trp) metabolism from dietary sources produces indole and its derivatives such as indoleacrylic acid (IA), indole acetic acid (IAA), indole‐3‐aldehyde (IAld), indole‐3‐propionic acid (IPA), and indole lactic acid (ILA) serving as endogenous AhR ligands that are able to enhance host immunity and metabolic hemostasis.^[^ ^23^, ^24^, ^25^ ^]^ Previous both pre‐clinical and clinical studies showed that decreased endogenous AhR agonists profoundly facilitated the development of metabolic syndrome such as high blood pressure, diabetes, and obesity.^[^ ^26^ ^]^ Hence supplementation with Trp metabolites acting as AhR endogenous ligands has been used to protect against some metabolic diseases such as inflammatory bowel diseases (IBD) and colitis by maintaining gut barrier function.^[^ ^27^ ^]^ Administration of indoles and its derivative indole‐3 aldehyde (I3A) promotes epithelial cells proliferation, thus exhibiting prolongation of life.^[^ ^13^ ^]^ Despite the close relationship between AhR and intestinal homeostasis, AhR‐mediated gut‐bone signaling pathway involved in physiological process of metabolic diseases has not yet been fully explored.

The Wnt/β‐catenin signaling pathway contributes immensely to stem cell proliferation and development, and tissue homeostasis.^[^ ^28^, ^29^, ^30^ ^]^ In the Wnt signaling cascade, β‐catenin (also called *Cbnnt1*) responsible for signal transduction to the nucleus^[^ ^31^ ^]^ is a structural component of E‐cadherin serving as one of AJ proteins.^[^ ^31^, ^32^ ^]^ Notably, Wnt/β‐catenin has been shown to be essential for intestinal stem cells differentiation and gut barrier function.^[^ ^33^ ^]^ More interestingly, growing evidence suggests the existence of crosstalk between Wnt signaling and AhR.^[^ ^34^ ^]^ AhR itself was reported as a target gene of Wnt/β‐catenin pathway that has been proposed as a key regulator of hepatic cytochrome P450 expression.^[^ ^35^, ^36^, ^37^, ^38^ ^]^ Coordinated activation of AhR and β‐catenin signaling was reported to promote proliferation and metastasis of hepatocellular carcinoma cells via upregulation of indoleamine 2,3‐Dioxygenase 1 (IDO1).^[^ ^39^ ^]^ Based on these proposed physiological functions of both AhR and Wnt/β‐catenin pathway, we hypothesize that modulating the interplay of AhR and β‐catenin might be developed as a novel potential strategy for intervention of metabolic diseases via maintaining intestinal homeostasis.

In this study, our clinical cohort experiments revealed that postmenopausal individuals exhibited markedly lower levels of microbial Trp metabolites (IAA and IPA) than premenopausal individuals. Similar AhR ligands reduction was also observed in OVX‐induced osteoporosis mouse model. On the basis of these findings, we proposed this hypothesize that oral supplementation with IAA and IPA may effectively ameliorated bone loss. Given that gut‐bone signaling highly contributes to bone health, a combination of 16S rRNA gene sequencing, targeted HPLC‐QQQ‐MS‐based metabolomics and a series of biological assays was employed to investigate the underlying mechanisms by which Trp metabolites ameliorate bone loss via gut‐bone crosstalk in vivo and in vitro. These findings provide a novel strategy targeting cross‐talk between the gut and bone to improve osteoporosis.

## 2 Results

### 2.1 Altered Microbiota Composition and Reduced Trp Metabolism in Animal Models of Osteoporosis

**Figure 1:**
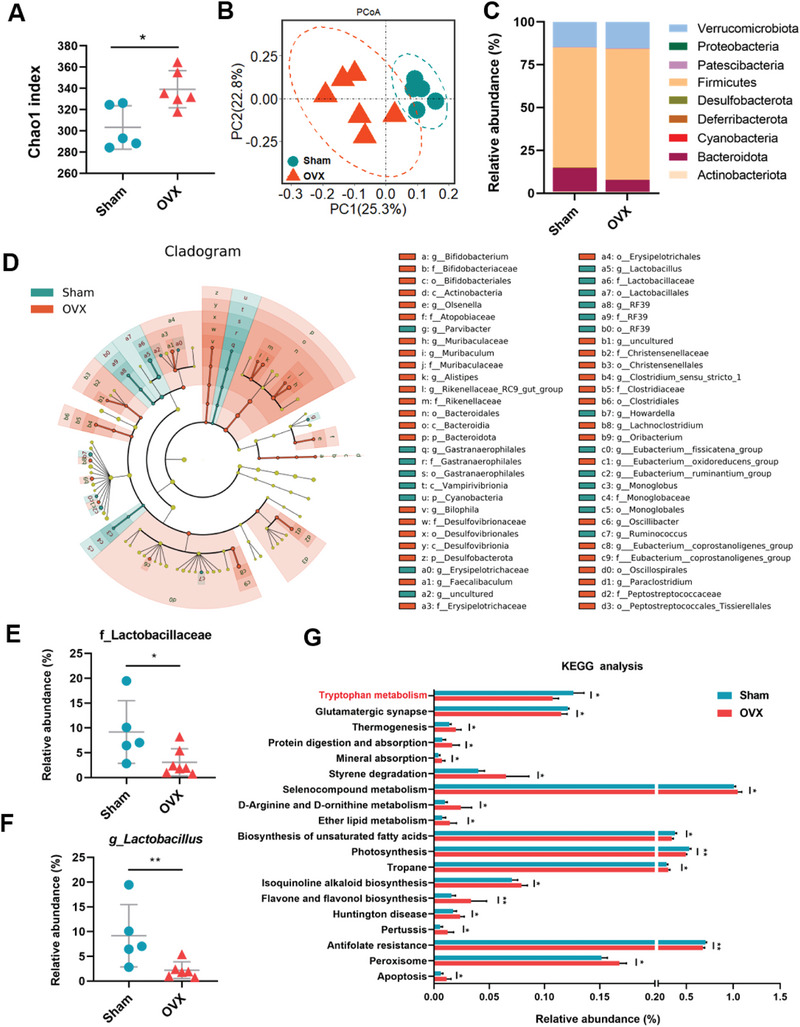
Altered microbiota composition and reduced Trp metabolism in animal models of osteoporosis. A) α‐diversity analysis based on numbers of features. B) β‐diversity index analysis based on Bray–Curtis dissimilarities (PCoA). C) Relative abundances of gut bacteria at the phylum level. D) Analysis of the relative abundance of taxa among different groups using a LEfSe cladogram. LDA effect size cladograms showing the taxa most differentially associated with Sham (green) or OVX mice (orange) (Wilcoxon rank‐sum test). Circle sizes in the cladogram plot are proportional to bacterial abundance. The circles represent, going from the inner to outer circle: genus, class, order, family, and phylum. LDA ≥ 2.0 and p ≤ 0.05 (Wilcoxon rank‐sum test) was regarded as significance. E) Relative abundance of family *Lactobacillaceae* (Sham, *n* = 5; OVX, *n* = 6). F) Relative abundance of genus *Lactobacillus* (Sham, *n* = 5; OVX, *n* = 6). G) PICRUSt2 findings with significant differences between Sham and OVX group. Data are shown as mean ± SD. P values were obtained by two‐tailed Student's *t*‐test, ^*^ *p* < 0.05, ^**^ *p* < 0.01, and ^***^ *p* < 0.001.

16S rRNA gene sequencing analysis showed that ovariectomy (OVX)‐induced postmenopausal osteoporosis mice exhibited significantly altered microbial community, manifested by marked different diversity and microbiota composition between the two groups (**Figure** **1**A–C). Specifically, OVX significantly upregulated Firmicutes and downregulated Bacteroidota in cecal contents of mice, which are dominant bacteria at the phylum level (Figure 1C). At the genus level, comparison between bacterial community characteristics was tabulated using a LEfSe cladogram representing the relative abundance of each genus (Figure 1D). The results suggested that OVX induced profound alterations in the microbiota composition (Figure 1D). Compared with Sham mice, OVX‐induced osteoporosis mice showed significant increases in the gut bacteria belonging to the *Muribaculaceae*, *Bifidobacteriaceae*, and *Clostridiaceae* families (Figure 1D). Especially, the relative abundances of family *Lactobacillaceae* and genus *Lactobacillus* were markedly downregulated in the cecal contents of OVX mice in comparison with Sham mice (Figure 1E,F).

To predict the relationship between abundance of gene families and functional pathways of microbial communities in the cecal contents, PICRUSt2 (phylogenetic investigation of communities by reconstruction of unobserved states) was employed based on the 16S rRNA gene sequencing and the Green Genes database (Figure 1G). The results of metagenomic analysis showed that OVX significantly altered many bacterial pathways involving in amino acid, lipid, mineral absorption, and apoptosis and so on. Of particular note was that OVX mice exhibited significantly reduced Trp metabolism that is highly associated with gut microbiota, the downstream metabolites, and metabolic diseases (Figure 1G). Spearman correlation analysis showed that the levels of indole metabolites such as IAA, IPA, and ILA were positively correlated to the relative abundance of *Lactobacillus* (Figure S1A–C, Supporting Information). To further verify the specific association between Trp metabolites and *Lactobacillus*, we have also performed direct supplementary experiments in which several *Lactobacillus* species were colonized to mice with and without antibiotics (ABX) treatment (Figure S1D, Supporting Information). Targeted metabolomics showed that the levels of indole and indole derivatives, especially IAA, in feces and colon of mice upon *Lactobacillus* supplementation were much higher than those of mice with only antibiotics treatment (Figure S1E, Supporting Information), suggesting that indole metabolites (IAA and IPA) are derived from microbes, especially at least *Lactobacillus*.

### 2.2 Impaired Microbial Trp Metabolites in Animal Models with Osteoporosis and Postmenopausal Individuals

**Figure 2:**
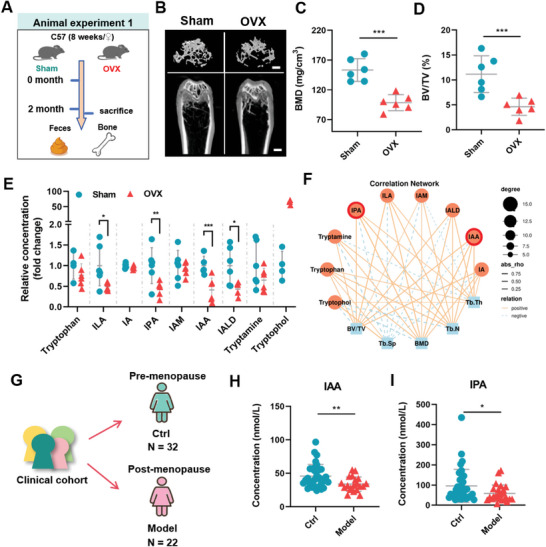
Impaired microbial AhR ligands in animal models of metabolic diseases with bone loss and postmenopausal individuals. A) Schematic illustration of the experimental flow in animal experiment 1. B) Representative µCT images and 3D reconstruction of trabecular bone (Scale bar = 200 µm). C, D) Quantitative analysis of 3D parameters for trabecular bone microarchitecture, including BMD and BV/TV (*n* = 6). E) Relative concentration of fecal AhR ligands. F) Correlation network between AhR ligands and trabecular bone microarchitecture parameters. G) Serum concentrations of IAA (H) and IPA (I) of individuals with premenopausal (Ctrl, *n* = 32) and post‐menopausal (Model, *n* = 22) women. Data are shown as mean ± SD. P values were obtained by two‐tailed Student's *t*‐test, ^*^ *p* < 0.05, ^**^ *p* < 0.01, and ^***^ *p* < 0.001.

To investigate the relevance of microbial Trp metabolites to metabolic diseases with bone loss, we quantitatively analyzed the concentrations of specific Trp metabolites in fecal samples of several animal models with OVX‐induced postmenopausal osteoporosis (**Figure** **2**A; Figure S2, Supporting Information). Significant bone loss was observed in OVX‐induced osteoporosis mouse model (Figure 2B), manifested by marked reduction of bone mineral density (BMD) and bone volume/tissue volume ratio (BV/TV) (Figure 2C,D). Targeted UHPLC‐QQQ‐MS‐based metabolomics analysis indicated that much lower concentrations of AhR ligands such as indole derivatives (IAA and IPA) were observed in fecal samples from animal models with metabolic diseases than those of their corresponding controls (Figure 2E). Interestingly, spearman correlation analysis showed that the levels of indole metabolites, especially IAA and IPA, exhibited markedly positive correlation with parameters of bone loss including BMD, BV/TV, trabecular thickness (Tb.Th), and trabecular number (Tb.N) (Figure 2F). Individuals displaying postmenopausal bone loss, similarly showed lower serum concentrations of IAA and IPA, microbial AhR ligands, than premenopausal individuals (Figure 2G–I). Collectively, these results demonstrate that OVX‐induced bone loss is highly relevant to significantly reduced microbiota‐derived Trp metabolites.

### 2.3 Supplementation with Microbial Trp Metabolites Effectively Ameliorates OVX‐Induced Bone Loss in an Intestinal AhR‐Dependent Manner

**Figure 3:**
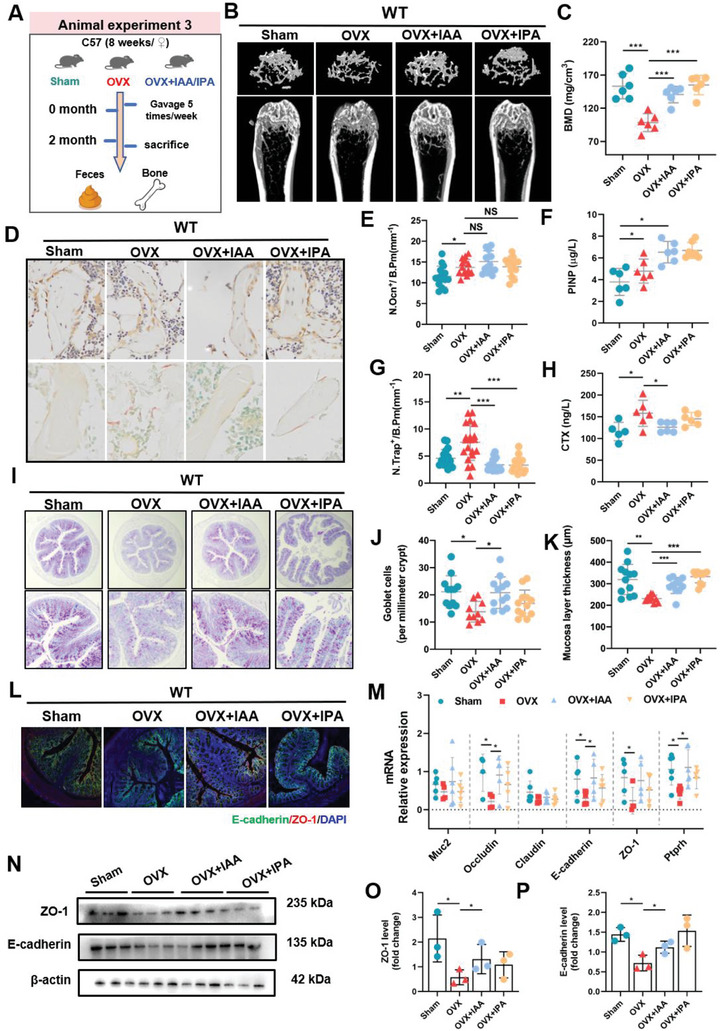
Supplement with IAA and IPA alleviate OVX‐induced osteoporosis in WT mice. A) Schematic illustration of the experimental flow in animal experiment 4. B) Representative µCT images and 3D reconstruction of trabecular bone in WT mice (Scale bar = 200 µm). C) Bone mineral density (BMD) of WT mice (*n* = 6). D) Representative images of immunohistochemistry staining and TRAP staining of distal femur (scale bar = 50 µm, *n* = 3). E) Analysis of Ocn‐positive cell number and G) TRAP‐positive cell number per bone surface. Three slices with five regions in each slice were analyzed for distal femur of mice. Each dot indicates one region (*n* = 3). F) ELISA of serum P1NP reflecting the bone formation (*n* = 6). H) ELISA of serum CTX‐1 reflecting the bone resorption (*n* = 6). I) Microscopic images of periodic acid‐Schiff (PAS) stains of colons (Scale bar = 100 µm, *n* = 3). J) The number of goblet cells in colon. Three slices of colonic samples of each mouse were analyzed with 4 points in each slice. Each dot indicates one region (*n* = 3). K) Crypt length of mice, each dot indicates one region (*n* = 3). L) Representative confocal images of E‐cadherin (green), ZO‐1 (red) and DAPI (blue) in immunostained sections (Scale bar = 100 µm, *n* = 3). M) Ileac mRNA levels of *Muc2*, *Occludin*, *Claudin*, *E‐cadherin*, *ZO‐1*, and *Ptprh*. N) Representative western blots of ZO‐1 and E‐cadherin expression in colon tissues (*n* = 3). O, P) Quantitative analysis of ZO‐1 and E‐cadherin proteins (*n* = 3). Data are shown as mean ± SD. P values were obtained by one‐way ANOVA with multiple comparisons, ^*^ *p* < 0.05, ^**^ *p* < 0.01, and ^***^ *p* < 0.001.

**Figure 4:**
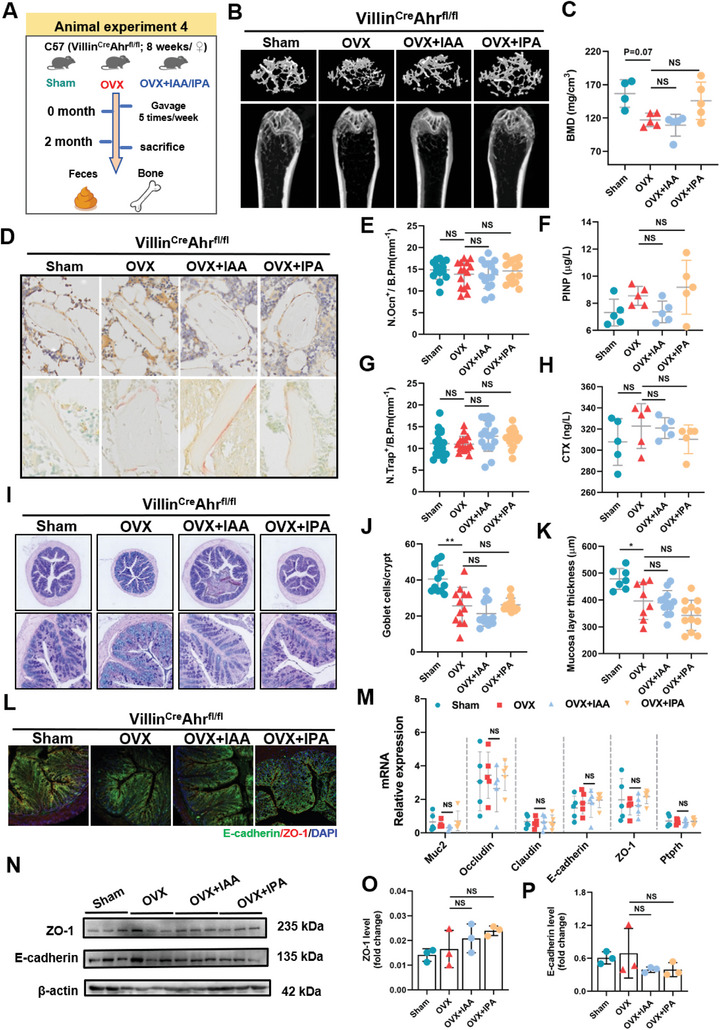
Supplement with IAA and IPA could not alleviate OVX‐induced osteoporosis in Villin^Cre^Ahr^fl/f^ mice. A) Schematic illustration of the experimental flow in animal experiment 5. B) Representative µCT images and 3D reconstruction of trabecular bone in Villin^Cre^Ahr^fl/fl^ mice (Scale bar = 200 µm). C) Bone mineral density (BMD) of Villin^Cre^Ahr^fl/fl^ mice (*n* = 5). D) Representative images of immunohistochemistry staining and TRAP staining of distal femur (scale bar = 50 µm, *n* = 3). E) Analysis of Ocn‐positive cell number and G) TRAP‐positive cell number per bone surface. Three slices with five regions in each slice were analyzed for distal femur of mice. Each dot indicates one region (*n* = 3). F) Serum P1NP reflecting bone formation (*n* = 5). H) Serum CTX‐1 reflecting the bone resorption detected by ELISA (*n* = 5). I) Microscopic images of periodic acid‐Schiff (PAS) stains of colons (Scale bar = 100 µm, *n* = 3). J) The number of goblet cells in colon. Three slices with four points in each slice were analyzed for colonic samples of each mouse. Each dot indicates one region (*n* = 3). K) Crypt length in mice was measured by mucosa layer thickness. Each dot indicates one region (*n* = 3). L) Representative confocal images of E‐cadherin (green), ZO‐1 (red), and DAPI (blue) immunostained sections (Scale bar = 100 µm, *n* = 3). M) Ileac mRNA levels of *Muc2*, *Occludin*, *Claudin*, *E‐cadherin*, *ZO‐1*, and *Ptprh*. N) Representative western blots (*n* = 3) of ZO‐1 and E‐cadherin expression in colon tissues. O, P) Quantitative analysis of ZO‐1 and E‐cadherin (*n* = 3). Data are shown as mean ± SD. P values were obtained by one‐way ANOVA with multiple comparisons, ^*^ *p *< 0.05, ^**^ *p *< 0.01, and ^***^ *p *< 0.001.

We next used the typical OVX‐induced osteoporosis mouse model to explore whether and/or how dietary treatment with microbial Trp metabolites can improve bone loss. µCT imaging and quantitative analysis showed that supplementation with IAA and IPA (20 mg kg^−1^ body weight) by gavage for 10 weeks markedly improved OVX‐induced bone loss of wild type (WT) mice (**Figure** **3**A), shown with significant upregulation of BMD and BV/TV (Figure 3B; Figure S3A, Supporting Information) and Tb. Th (Figure S3B, Supporting Information) together with significant downregulation of trabecular separation (Tb. Sp) (Figure S3C, Supporting Information). Furthermore, immunohistochemical osteocalcin (OCN) staining showed that supplementation with IAA and IPA markedly increased the number of osteoblasts on trabecular bone surface of OVX WT mice (Figure 3D,E). Enzyme‐linked immunosorbent assay (ELISA) revealed that supplementation with IAA and IPA further enhanced serum level of the N‐terminal propeptide of type I procollagen (PINP), a biomarker of osteogenic differentiation (Figure 3F). In addition, quantitative measurement coupled with tartrate‐resistant acid phosphatase (TRAP) staining suggested that treatment with IAA markedly decreased the levels of Trap positive (N. Trap^+^) and C‐telopeptide of type I collagen (CTX‐I), typical biomarkers of osteoclast activity and bone resorption, in OVX WT mice (Figure 3G,H). Most notably, these ameliorative effects of IAA and IPA treatments on bone loss were not significantly observed in OVX mice with intestinal AhR knockout (Villin^Cre^Ahr^fl/fl^) (**Figure** **4**A–H; Figure S3D–F, Supporting Information), suggesting that supplementation with IAA and IPA has a potential role in ameliorating OVX‐induced bone loss in an intestinal AhR‐dependent manner.

### 2.4 Supplementation with Microbial Trp Metabolites Restores OVX‐Induced Intestinal Barrier Integrity in an Intestinal AhR‐Dependent Manner

Given that intestinal AhR was found to be closely associated with OVX‐induced bone loss, we next investigated the effects of supplementation with microbial Trp metabolites on intestinal barrier function. Intestinal AB‐PAS staining showed that supplementation with IAA and IPA effectively restored intestinal structural integrity that was altered in OVX mice (Figure 3I), quantitatively manifested by marked elevation in the number of goblet cells (Figure 3J) and mucosa layer thickness (Figure 3K). Similar to previous studies reporting intestinal barrier integrity in OVX mice,^[^ ^40^, ^41^ ^]^ we found that OVX mice exhibited lower protein and mRNA levels of ZO‐1 and E‐cadherin (Figure 3L–P), junction components at the apical site of epithelial cell, together with lower mRNA level of protein tyrosine phosphatase receptor type H gene (*Ptprh*), a typical marker of gut permeability relevant to intestinal barrier function (Figure 3M), than those of Sham WT mice. Supplementation with IAA and IPA strikingly restored the levels of intestinal barrier function‐related biomarkers like upregulation of junction proteins between epithelial cells of OVX WT mice (Figure 3L–P). However, supplementation with IAA and IPA did not exhibit intestinal repairing capacity in OVX mice with intestinal AhR knockout, shown with no significant changes in the gut structural integrity‐related parameters including the number of goblet cells (Figure 4J), mucosa layer thickness (Figure 4K), protein and mRNA levels of junction components and gut permeability (Figure 4L–P). These findings suggest that supplementation with IAA and IPA can effectively restore the gut barrier function of OVX mice with bone loss via activation of intestinal AhR.

### 2.5 Activation of AhR Repairs Intestinal Barrier Function via Regulating Wnt/β‐catenin Signaling Pathway

**Figure 5:**
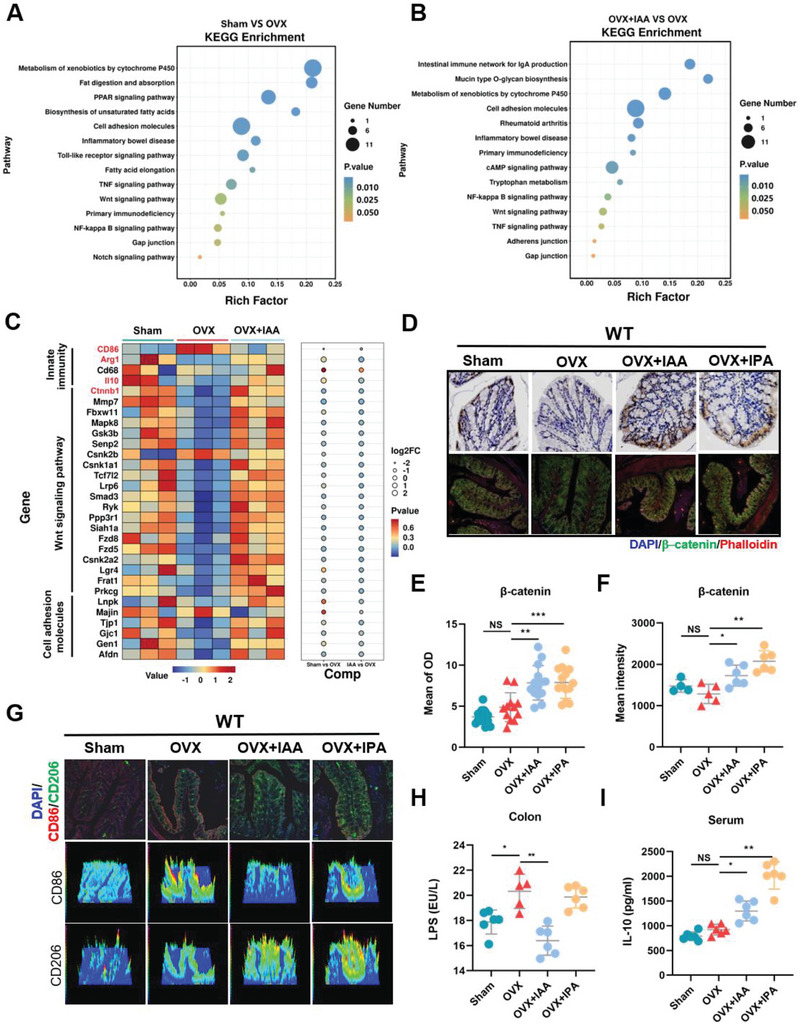
Transcriptomic analysis showed that activation of AhR repairs intestinal barrier function via regulating Wnt/β‐catenin signaling pathway. RNA sequence analysis of KEGG enrichment in Sham versus OVX (A), OVX+IAA versus OVX (B). C) Expression profiles of innate immunity, Wnt signaling pathway, and cell adhesion molecules in the group of Sham, OVX, and OVX+IAA. D) Colon immunohistochemistry staining and immunofluorescence confocal images of β‐catenin (green), Phalloidin (red), and nuclei (blue) in WT mice (Scale bar = 100 µm). E,F) Quantitative of immunohistochemistry and IF images by mean of OD and mean intensity, each dot indicates one region (*n* = 3). G) immunofluorescence confocal images of M1 (CD86, red), M2 (CD206, green), and nuclei (DAPI, blue). And 3D profiles of expression intensity of CD86 and CD206 among groups (Scale bar = 100 µm). H) ELISA analysis of colonic LPS (*n* = 6). I) ELISA analysis of serum IL‐10 (*n* = 6). Data are shown as mean ± SD. P values were obtained by one‐way ANOVA with multiple comparisons, ^*^ *p* < 0.05, ^**^ *p* < 0.01, and ^***^ *p* < 0.001.

**Figure 6:**
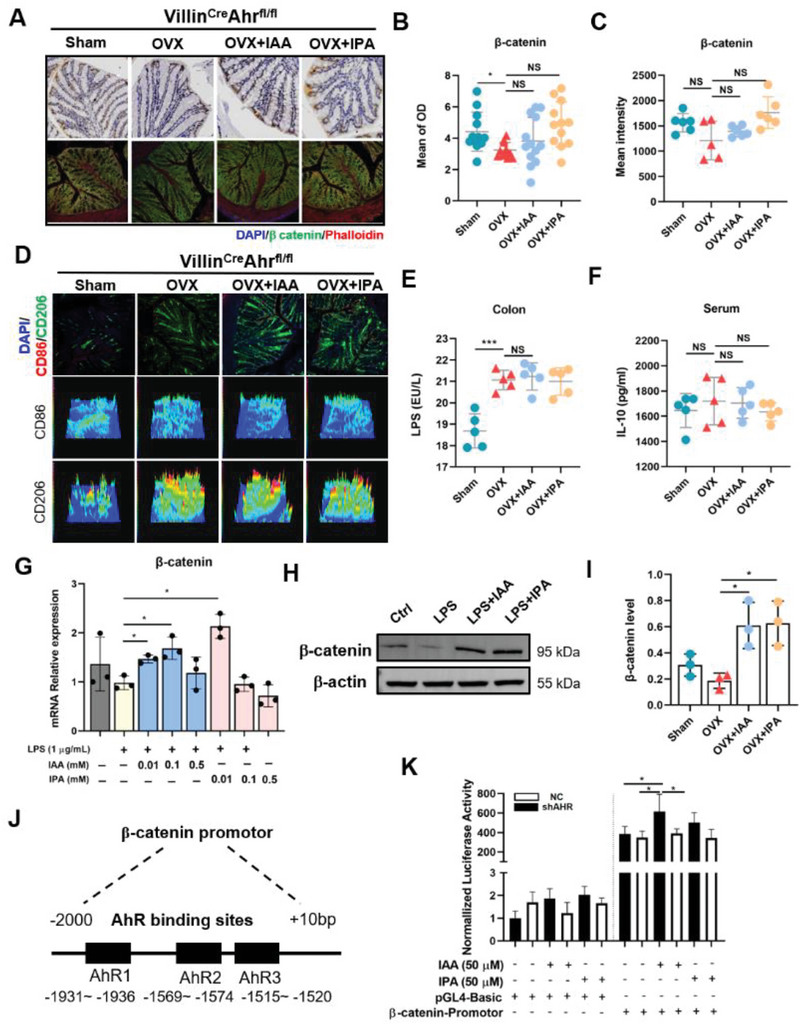
Activation of AhR by supplement with IAA and IPA regulates macrophage phenotypes and interacts with β‐catenin promotor. A) Colon immunohistochemistry staining and immunofluorescence confocal images of β‐catenin (green), Phalloidin (Red), and nuclei (blue) in Villin^Cre^Ahr^fl/fl^ mice (Scale bar = 100 µm). B,C) Quantitative of immunohistochemistry and immunofluorescence images by mean of OD and mean intensity, each dot indicates one region (*n* = 3). D) Immunofluorescence confocal images of M1 (CD86, red), M2 (CD206, green), and nuclei (DAPI, blue) (Scale bar = 100 µm). E) ELISA analysis of colonic LPS (*n* = 5). F) ELISA analysis of serum IL‐10 (*n* = 5). G) mRNA level of β‐catenin in Caco‐2 cells treated with IAA and IPA with different dosages. H) Protein level of β‐catenin in Caco‐2 cells treated with LPS and LPS plus IAA/IPA. I) Quantitative analysis of β‐catenin protein (*n* = 3). J) Schematic of β‐catenin promotor construct of *AhR* gene. AhR‐binding sites are presented spanning from −1931 to −1936 bp, −1569 to −1574 bp, and −1515 to −1520 bp. K) Dual luciferase assay in 293T cells with negative control (NC) and *AhR* gene knockdown (shAhR) stimulated by IAA and IPA. Data are shown as mean ± SD. P values were obtained by one‐way ANOVA with multiple comparisons, ^*^ *p* < 0.05, ^**^ *p* < 0.01, and ^***^ *p* < 0.001.

To explore the molecular mechanisms by which AhR activation regulates epithelial barrier integrity, we analyzed RNA sequencing profiles in intestine obtained from Sham and OVX WT mice with and without IAA treatment. PCA analysis of RNA sequencing revealed clear separation between different groups and robust clustering of samples within each group (Figure S4A, Supporting Information). Differential gene expression analysis showed that supplementation with IAA induced marked upregulation of 477 genes and downregulation of 264 genes in intestine of OVX mice (*p* < 0.05 and fold change > 1.5) (Figure S4B,C, Supporting Information). Especially, Kyoto encyclopedia of genes and genomes (KEGG) enrichment analysis highlighted several signaling pathways including AhR‐related metabolism of xenobiotics by cytochrome, Wnt signaling pathway, cell adhesion molecules, calcium signaling pathway, and innate immunity (**Figure** **5**A,B). Transcriptomics analysis further suggested that these differentially expressed genes induced by IAA treatment were mainly originated from activation of immune response, Wnt signaling pathway and cell adhesion molecules (Figure 5C). Notably, gene set enrichment analysis (GSEA) revealed that intestinal Wnt signaling pathway was inhibited in OVX mice and subsequently activated upon IAA treatment (Figure S4D,E, Supporting Information). Based on the analysis of differentially expressed genes, we found that IAA treatment markedly upregulated mRNA levels of *Arg1*, *Il‐10*, and *β‐catenin* (*Ctnnb1*) and downregulated mRNA levels of *CD86* that are involved in innate immunity and Wnt signaling pathway in OVX mice (Figure 5C). Furthermore, qualitative and quantitative analyses with immunohistochemistry and immunofluorescence showed that supplementation with IAA and IPA significantly repaired intestinal epithelium integrity and upregulated the level of *β‐catenin* in intestine of OVX WT mice (Figure 5D–F). Meanwhile, supplementation with IAA and IPA markedly decreased serum LPS accompanied by significant elevation in the level of serum IL‐10 in OVX WT mice (Figure 5H,I). However, these alterations in the levels of *β‐catenin*, LPS and IL‐10 were not observed in OVX mice with intestinal AhR knockout (**Figure** **6**A–F).

We next used LPS‐treated Caco‐2 cells acting as in vitro model with intestinal disorder to further explore the roles of AhR and Wnt/β‐catenin signaling pathway in epithelial cells. Interestingly, LPS treatment markedly decreased the protein and mRNA levels of β‐catenin that were significantly reversed by IAA and IPA treatments (Figure 6G–I). IAA treatment also markedly increased mRNA level of *Ahr*, and *ZO‐1* that were decreased by LPS exposure in Caco‐2 cells (Figure S5A,B, Supporting Information). Importantly, we discovered that at least three binding sites of β‐catenin promotor from −2000 to +10 bp interacts with AhR protein via simulation of the JASPAR database (Figure 6J; Figure S6, Supporting Information). To experimentally validate AhR binding to β‐catenin promotor, we constructed a pGL4‐Basic‐β‐catenin‐promotor‐luc plasmid spanning from −2000 to +10 bp of β‐catenin promotor cloned upstream of luciferase in HEK 293T cells (Figure 6J). After transfection of β‐catenin luciferases reporter vectors into HEK 293T cells with and without AhR knockdown (Figure S5C, Supporting Information), we found that only IAA administration induced a marked increase in the luciferase activity of β‐catenin reporter in HEK 293T cells, whereas no significant change was observed in *Ahr*‐knockdown HEK 293T cells upon IAA supplementation (Figure 6K). Taken together, these findings reveal that activation of AhR by supplementation with IAA repairs intestinal barrier function via regulating Wnt/β‐catenin signaling pathway.

### 2.6 IL‐10 Secreted by M2 Macrophage Directly Interacts with Osteocytes

Given that gut‐bone signaling axis highly contributes to bone health, we next explore the crosstalk between the gut and bone. In OVX WT mice, OVX operation induced significant upregulation of M1 macrophages (CD86^+^) and downregulation of M2 macrophages (CD206^+^) due to LPS‐induced inflammatory status in intestine (Figure 5C,G). As a result, mRNA level of *Il‐10* secreted by M2 macrophage was consequently decreased in intestine of OVX WT mice compared with Sham mice (Figure 5C). Supplementation with IAA and IPA led to the switching from M1 to M2 activated macrophage phenotypes, manifested by a marked reduction in CD86^+^ macrophages and a substantial increase in CD206^+^ macrophages in intestinal lamina propria of OVX mice (Figure 5C,G). Consequently, supplementation with Trp metabolites, especially IAA, markedly downregulated mRNA level of intestinal *Il‐10* (Figure 5C) and upregulated IL‐10 level in serum (Figure 5I), suggesting that supplementation with IAA promoted expansion of IL‐10 from intestine to blood circulation. Interestingly, no significant changes in the switch of macrophage phenotypes and the level of serum IL‐10 were observed in OVX mice with intestinal AhR knockout upon IAA and IPA supplementation (Figure 6D,F).

**Figure 7:**
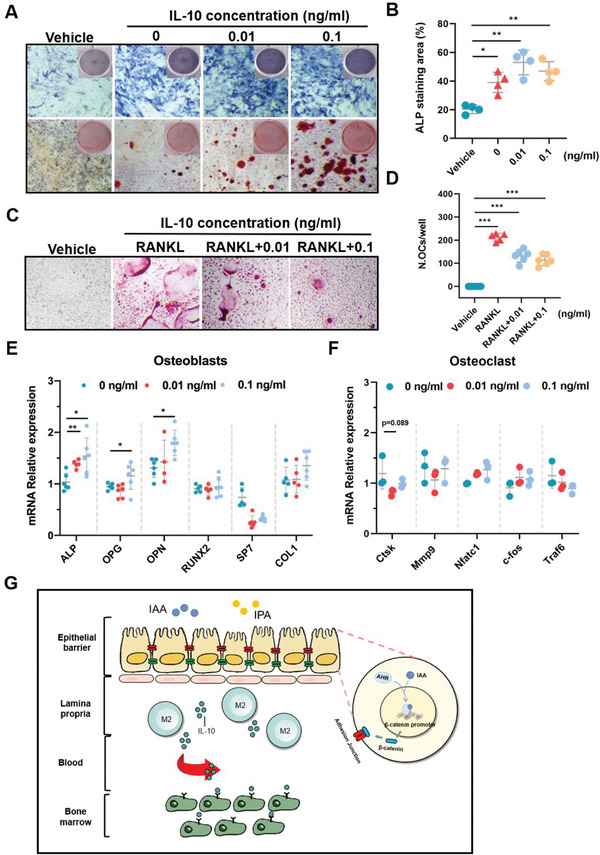
IL‐10 promotes osteoblastogenesis and inhibits osteoclastogenesis. A) ALP and Alizarin red staining for primary osteoblasts treated with 0, 0.01, and 0.1 ng mL^−1^ IL‐10 (scale bar = 50 µm). B) Quantitative analysis of ALP staining areas. C) Primary BM macrophages were incubated with M‐CSF (25 ng mL^−1^) and RANKL (50 ng mL^−1^) and treated with IL‐10 at 0, 0.01, and 0.1 ng mL^−1^ (Scale bar = 50 µm). D) The number of TRAP positive (purple, cytoplasm) multinucleated osteoclasts in each well (*n* = 6). E) mRNA levels of *Alp*, *Opg*, *Opn*, *Runx2*, *Sp7*, and *Col1* in osteoblasts. F) mRNA levels of *Ctsk*, *Mmp9*, *Nfatc1*, *C‐fos*, and *Traf6* in osteoclasts. G) Summary of molecular mechanisms by which IAA and IPA ameliorate OVX‐induced osteoporosis via activating AhR‐mediated gut‐bone signaling pathway. Data are shown as mean ± SD. P values were obtained by one‐way ANOVA with multiple comparisons, ^*^ *p* < 0.05, ^**^ *p* < 0.01, and ^***^ *p* < 0.001.

To verify the direct effects of IL‐10 on osteoblasts and osteoclasts, we further examine cell proliferation and differentiation of osteoblasts and osteoclasts under the condition of IL‐10 administration in vitro. We found that IL‐10 exposure dose‐dependently promoted osteoblast differentiation and formation (**Figure** **7**A,E) and inhibited RANKL‐induced osteoclastogenesis (Figure 7C,F). Specifically, IL‐10 exposure induced marked upregulation of alkaline phosphatase (ALP) activity and mineralization nodules formation (Figure 7A,B), suggesting IL‐10‐induced osteoblastogenesis. Supportive evidence can be found from markedly elevated osteogenesis‐related genes including *Alp*, *Opg*, and *Opn* in a dose‐dependent manner (Figure 7E). TRAP staining for osteoclasts also showed that IL‐10 treatment dose‐dependently prevented osteoclasts differentiation (Figure 7C), shown with a marked reduction of the number of TRAP‐positive multinucleated osteoclasts (N. OCs) (Figure 7D), whereas no significant changes were observed in the mRNA levels of osteoclastogenesis‐related genes including *Ctsk*, *Traf6*, and *Mmp9* (Figure 7F). Collectively, these in vivo and in vitro results suggest that supplementation with IAA and IPA promotes secretion and expansion of IL‐10 from intestinal M2 macrophage to bone marrow, thus simultaneously regulating osteoblast and osteoclast differentiation.

## 3 Discussion

Aging and aging‐related diseases are often accompanied by bone loss at least partially due to decreased immunity and estrogen levels.^[^ ^42^ ^]^ Growing evidence has shown that aging and aging‐related diseases with bone loss also highly attribute to disruption of the gut microbiota and its metabolites.^[^ ^40^ ^]^ Herein OVX mice exhibited marked alteration in the gut microbial community, especially reduced family *Lactobacillaceae* and genus *Lactobacillus*, which are consistent with significant downregulation of Trp metabolism in OVX mice. Targeted quantification of Trp metabolites revealed that postmenopausal individuals and animal models of metabolic diseases such as osteoporosis, obesity and aging exhibited significant reduction of microbiota‐derived Trp metabolites, especially IAA and IPA. Importantly, these markedly reduced endogenous AhR ligands were also found to positively correlate with bone loss in animal models of metabolic diseases. It is therefore anticipated that supplementation with IAA and IPA may ameliorate bone loss along with improvement of metabolic diseases. Actually, supplementation with Trp metabolites has been shown to protect mice from ethanol‐induced steatohepatitis^[^ ^43^ ^]^ and inflammatory bowel diseases (IBD) by reducing gut permeability via activation of AhR.^[^ ^16^ ^]^ In this study, typical OVX mice models with and without intestinal AhR knockout were employed to explore the underlying mechanisms by which IAA and IPA treatments improve bone loss via regulating gut‐bone signaling axis (Figure 7G).

From the perspective of osteoporosis phenotypes, supplementation with IAA and IPA effectively ameliorated bone loss of OVX mice in an intestinal AhR‐dependent manner. Mechanistically, intestinal AhR activation by IAA and IPA supplementation effectively repaired intestinal barrier integrity by stimulating Wnt/β‐catenin signaling pathway. Consequently, IAA and IPA supplementation switched M1 macrophage to M2 macrophage, releasing large amount of IL‐10 that expanded from intestinal lamina propria to bone marrow, thereby promoting osteoblastogenesis and inhibiting osteoclastogenesis. It is well‐documented that intestinal AhR plays key roles in mediating host immune response and gut barrier integrity. AHR deficiency in intestinal epithelial cells (IECs) was reported to promote inflammation and colon tumorigenesis in mice.^[^ ^44^ ^]^ Previous studies showed that three Trp metabolites such as indole‐3‐ethanol, indole‐3‐pyruvate, and indole‐3‐aldehyde derived from gut microbiota protected against IBD by regulating gut barrier function in an AhR‐dependent manner.^[^ ^16^ ^]^ Notably, Wnt/β‐catenin pathway is also important for intestinal stem cells (ISCs) proliferation and differentiation that are highly related to gut barrier function.^[^ ^45^ ^]^ Previous study reported that probiotic strain *Lactobacillus rhamnosus* GG (LGG) can promote bone formation and increase bone density in mice via producing microbial butyrate, which increased the production of the osteogenic Wnt ligand (Wnt10b) by CD8^+^ T cells to stimulate bone formation.^[^ ^46^ ^]^ Although both AhR and Wnt/β‐catenin participate in mediating gut barrier function, the interplay between them maintaining intestinal homeostasis and thereby improving bone loss remains elusive. In the current study, we demonstrate that intestinal AhR activation by IAA supplementation directly binds to β‐catenin promotor stimulating β‐catenin expression, thus effectively repairing intestinal barrier function in OVX mice. IAA supplementation exhibited negligible ameliorative effects on both intestinal homeostasis and bone loss of OVX mice with intestinal AhR knockout (Villin^Cre^Ahr^fl/fl^). These findings strongly suggest that IAA but not IPA supplementation ameliorates bone loss via regulating intestinal AhR‐Wnt/β‐catenin signaling pathway.

Increasing evidence has shown that gut‐bone axis closely contributes to bone health due to the expansion of immune and inflammation signaling from the gut to bone marrow. In OVX mice, markedly increased gut permeability resulted in intestinal leakage and translocation of microbial products such as LPS, thereby triggering systemic inflammation and immune stress. It was also reported that OVX induced expansion of intestinal Th17 cells to bone marrow, directly or indirectly leading to osteoclast formation and bone loss.^[^ ^21^ ^]^ In this study, supplementation with IAA and IPA effectively repaired the gut barrier integrity and significantly decreased the levels of LPS and M1 macrophage in OVX mice in an AhR‐dependent manner. More importantly, supplementation with IAA and IPA markedly increased the level of M2 macrophage secreting abundant IL‐10 that expanded from intestinal lamina propria to blood and bone marrow. Consequently, in vitro IL‐10 exposure directly interacted with osteocytes, thereby simultaneously promoting osteoblastogenesis and inhibiting osteoclastogenesis in a dose‐dependent manner. It has been shown that IL‐10 acting as a powerful immune mediator with strong anti‐inflammatory activity can reduce tissue damage induced by excess inflammation via targeting both innate and adaptive immune responses, thus maintaining gut homeostasis. Mice with IL‐10 deficiency (*Il‐10* ^−/−^) exhibited significant mechanical fragility and osteopenia of both cancellous and cortical bone.^[^ ^47^ ^]^ Furthermore, *Il‐10* ^−/−^ mice with colitis also displayed marked bone loss due to gut barrier dysfunction.^[^ ^47^ ^]^ Interestingly, administration of engineered IL‐10 by intravenous injection can effectively suppressed rheumatoid arthritis through restoring immune cell composition and suppressing activated macrophages in murine models.^[^ ^48^ ^]^ Previous studies showed that nanometer hydroxyapatite particles promoted bone repair by inducing M2 macrophage polarization and increasing IL‐10 production in vitro.^[^ ^49^ ^]^ Dietary intake of indole was reported to promote epithelial cell proliferation and goblet cell differentiation, thus maintaining gut homeostasis and extending healthy lifespan via AhR and IL‐10 signaling.^[^ ^13^ ^]^ Although macrophages are the main source of IL‐10, many immunocytes such as T cells and B cells also secrete IL‐10 exerting anti‐inflammation activity.^[^ ^50^, ^51^ ^]^ The relationship between immunocytes, IL‐10 and bone mass is therefore definitely worth exploring.

It is worth noting that IAA and IPA exhibited varying degrees of ameliorative effects on intestinal AhR‐mediated signaling and bone loss probably due to their different molecular functions. Specifically, here a dual‐luciferase reporter assay showed that HEK 293T cells with *Ahr*‐knockdown exhibited lower luciferase activity of β‐catenin promotor than normal HEK 293T cells upon IAA instead of IPA. To a certain extent, only supplementation with IPA even increased a little amount of BMD in OVX mice with intestinal AhR knockout. One of the reasons is that IPA is recognized as an endogenous ligand of pregnane receptor (PXR)^[^ ^52^ ^]^ serving maintenance of intestinal mucosa stability. Furthermore, IPA itself exerts anti‐inflammatory and antioxidant effects through circulation from intestinal epithelium into the blood and multiple organs.^[^ ^52^, ^53^ ^]^ Their different mechanisms by which IAA acts on the intestinal epithelium via AHR activation, while IPA may at least partially continue to function through PXR pathway, warrant further in‐depth investigation.

In summary, this study reveals a novel mechanistic link among microbial Trp metabolites, AhR‐Wnt signaling and bone loss. Supplementation with IAA and IPA effectively repairs gut barrier function via interplay between intestinal AhR and Wnt/β‐catenin. Enhancement of M2 macrophage by IAA and IPA in the intestinal lamina propria generates more IL‐10 entering the bone marrow, thereby promoting osteoblastogenesis and inhibiting osteoclastogenesis (Figure 7G). These findings demonstrate that intestinal AhR‐mediated gut‐bone axis could be a potential preventive and therapeutic target against bone loss.

## 4 Experimental Section

### Animal Experiments

Animal experimental procedures were reviewed and approved by the animal ethics committee of Innovation Academy for Precision Measurement Science and Technology, CAS (APM No: APM20029, China). In OVX mice models (animal experiment 1, 4, and 5), female wild‐type C57BL/6 mice (*n* = 32, 8‐week‐old) and intestinal AhR knockout (*Villin^Cre^Ahr^fl/fl^*) mice (*n* = 20, 8‐week‐old) were purchased from Charles River Co. Ltd (Beijing, China). All mice were housed under specific pathogen‐free conditions with 12‐h light cycle and fed sterilized food with autoclaved water ad libitum. After acclimation for 2 weeks, mice were supplied with IAA and IPA (20 mg kg^−1^ body weight) or vehicle by gavage 5 times per week.^[^ ^43^, ^54^, ^55^ ^]^ After treatment for 2 weeks, mice were ovariectomy under the condition of tribromoethanol anesthesia. Mice upon sham operation were regarded as control group. Food intake and body weight of mice were monitored and recorded once a week for 10 weeks. In the animal experiment with bacteria colonization (animal experiment 2), a total of 18 male wild‐type C57BL/6 (6‐week‐old) were obtained from Moubaili Biotechnology Co., Ltd. (Wuhan, China) and housed in SPF‐animal room. Mice were separated in three groups (*n* = 6) and raised with standard normal‐chow diet. Antibiotic treatment was a mixture of antibiotics (ABX: 1 mg mL^−1^ ampicillin, 0.5 mg mL^−1^ vancomycin, 1 mg mL^−1^ metronidazole, and 1 mg mL^−1^ neomycin) and dissolved in anaerobic PBS. 0.2% tryptophan solutions were prepared freshly and were changed twice a week. For the PBS and ABX group, mice were received PBS or antibiotic mixture solution for 4 weeks, and 0.2% tryptophan supplied in water began at the third week. For the *Lactobacillus spp*. supplementation groups, *Lactobacillus spp*. were thawed under anaerobic conditions and diluted with anaerobic PBS to a final concentration of 2 × 10^8^ viable c.f.u. per 0.1 mL. Mice were first treated with an antibiotic mixture solution twice a week for 2 weeks to remove microbiota, and then orally administered with *Lactobacillus spp*. by gavage for another 2 weeks. All mice were sacrificed under isoflurane anesthesia after fasting for 8 h at the end of corresponding experimental period. Biological samples including bone, cecal contents, plasma, liver, spleen, ileum, and colon were collected and immediately stored at −80 °C for later experiments.

### Clinical Cohort Study

The human cohort studies were approved by the Ethics Committee of Hubei Provincial Hospital of Traditional Chinese Medicine (No: HBZY2020‐C47‐01, China) in accordance with the World Medical Association's Declaration of Helsinki. Human subjects were selected from Hubei Provincial Hospital of Traditional Chinese Medicine (Wuhan, China) and provided informed consent from all participants. Serum of healthy female individuals was collected and categorized into premenopausal (*n* = 32) and postmenopausal cohorts (*n* = 22) based on their ages (50 years old) and clinical menopausal diagnosis. For all subjects, fasting was required for 12 h prior to sampling. Targeted quantification of tryptophan metabolites in serum was performed using UHPLC‐QQQ‐MS descripted in detail as below.

### 16S rRNA Gene Sequencing and PICRUSt2 of Gut Microbiota

The total DNA of gut bacteria was extracted from mouse cecal contents (∼100 mg) for 16S rRNA gene sequencing analysis. The 16S rRNA gene PCR process used 338F_806R primers targeting the V3‐V4 region (Table S2, Supporting Information) and was amplified with a KAPA HiFiHotStart PCR Kit (KAPA Biosystem, USA). Paired‐end 2 × 300 bp reads were sequenced on an Ilumina MiSeq platform by Shanghai Majorbio Bio‐pharm Technology Co., Ltd. Prior to deblurring, sequencing reads were processed using QIIME 2 (version 2020.11). Additionally, R packages were utilized to visualize both α‐ and β‐diversity. The taxonomy of OTUs was determined using the SILVA_134 database. Functional predictions were derived from 16S rRNA sequences employing PICRUSt2^[^ ^56^ ^]^ (Phylogenetic Investigation of Communities by Reconstruction of Unobserved States 2), and subsequently annotated with the KEGG (Kyoto Encyclopedia of Genes and Genomes) database.

### µCT Analysis

Mice femurs were fixed in 4% paraformaldehyde for 2 days and stored in ethanol solution (70%). High‐resolution micro‐computed tomography (µCT) imaging was performed using NanoScan 1172 (Mediso). Skyscan (1276; Bruker). High‐resolution µCT scanner were set to 55 kVp and 200 µA. For the femoral trabecular region, 100 slices were analyzed, beginning 50 slices below the distal growth plate. 3D structure and morphometry were constructed and analyzed for bone volume/tissue volume ratio (BV/TV), bone mineral density (BMD), trabecular number (Tb.N), trabecular thickness (Tb.Th), and trabecular separation (Tb.Sp) in CTvol and Data‐viewer (Bruker Belgium).

### In Vitro Experiments

Cells were cultured in a humidified 5% CO_2_ incubator at 37 °C. Primary calvarial osteoblasts were isolated from newborn C57BL/6 mouse and bone marrow macrophages were isolated from 8‐week‐old C57BL/6 mice as previously published protocol.^[^ ^57^ ^]^ Caco‐2 cells (Procell Life Science & Technology Co, Ltd.) and 293T cells were cultured in MEM basic medium (Gibco) with 10% FBS and 1% penicillin‐streptomycin. For plasmid infection, the cells were selected with puromycin for 2 days with 3 µg mL^−1^. *AhR* knockdown in 293T cell was confirmed via qPCR and western blot analysis. In vitro experiments including treatments with LPS, Trp metabolites, and IL‐10 (Sino Biological) were performed.

### Targeted Quantification of Tryptophan Metabolites

Targeted quantification of Trp metabolites in feces of OVX mice models were conducted by ultrahigh‐performance liquid chromatograph (Agilent 1290) coupled with a model 6460 triple‐quadrupole mass spectrometer (UHPLC‐QQQ‐MS; Agilent Technologies, Inc.) in multiple‐reaction monitoring (MRM) mode. Data were analyzed via integral peak area by quantitative analysis program (Agilent). The procedures of sample preparation and LC‐MS detection were provided as previous publication.^[^ ^58^ ^]^

### RNA‐Seq and Transcriptomic Analysis

For RNA‐seq, TRIzol Reagent (Plant RNA Purification Reagent for plant tissue) was used to extract the total RNA from colon tissues according to the manufacturer's instructions (Invitrogen). RNA integrity was determined by Bioanalyzer (Agilent 2100) and quantified using the ND‐2000 (NanoDrop Technologies). Only high‐quality RNA samples RIN ≥ 6.5 were used for the subsequent experiments. The libraries were prepared following TruSeq RNA sample preparation Kit (Illumina, San Diego, CA). Next, paired‐end reads were sequenced with Illumina HiSeq Xten/NovaSeq6000 (2 × 150 bp read length). The following data was analyzed on Majorbio according to the standard protocols. Briefly, KEGG pathway enrichment analysis was performed using KOBAS (http://kobas.cbi.pku.edu.cn/home.do). Multiple testing was conducted using the Benjamini‐Hochberg (FDR) method, with a p‐value threshold of ≤ 0.05 to define significantly different KEGG pathways. Gene set enrichment analysis (GSEA) was employed to compare vehicle‐ and IAA‐treated mice using GSEA software (http://www.broad.mit.edu/GSEA).

### Dual‐Luciferase Reporter Assay

293T cells with *Ahr*‐knockout were seeded in a 24‐well plate (Table S1, Supporting Information). For β‐catenin activity analysis, cells were transfected with either a control vector (pGL4‐basic) or a vector harboring the β–catenin promoter (pGL4‐Basic‐β–catenin‐promoter‐luc). The cells were co‐transfected with a Renilla luciferase vector pRL‐TK as an internal control using Lipofectamine 3000 according to the manufacturer's protocol. The medium was replaced by fresh medium or containing IAA and IPA (50 ng mL^−1^) after 6 h. After 48 h transfection, the dual‐luciferase activity of β‐catenin was measured using a dual luciferase reporter gene assay kit II (Beyotime Biotechnology). The results were normalized to activity of the pRL‐TK control group.

### Alcian Blue and Periodic Acid Schiff's (AB‐PAS) Staining

Colon tissues of mice were immediately fixed in 4% paraformaldehyde solution. Colon tissues were then embedded in paraffin wax and sectioned 4 mm. Following de‐waxing in xylene and re‐hydrating through ethanol gradients, paraffin sections were stained and mounted for Alcian blue‐periodic acid Schiff (AB‐PAS). Images were taken by Olympus BX51 microscope and analyzed by Image J software (1.52v; USA). The number of goblet cells per unit length were counted by Image‐Pro Plus 6.0.

### Immunofluorescence

Fresh colon tissues of mice were fixed in 4% paraformaldehyde solution for 24 h, and then dehydrated, paraffin‐embedded and sectioned 3–4 µm. The sections were incubated with primary antibodies including ZO1 (1:1000; 21773‐1‐AP; Proteintech), E‐cadherin (1:200; 14472S; Cell Signaling), CD86 (1:500; Invitrogen), CD206 (1:500; Invitrogen), and β‐catenin (1:200; Proteintech). Corresponding secondary antibody was marked with appropriate species‐specific Alexa Fluor 488 antibodies (1:1000; A‐10680; ThermoFisher) and Alexa Fluor 647 Phalloidin (1:500; Solarbio) in 5% BSA in PBS in the dark at room temperature for 1 h. Nuclei were counterstained with DAPI (S2110; Solarbio). Three slices of colonic samples of each mouse were analyzed with 4 points in each slice. Thus, a total of 12 data points was statistically obtained. Nikon A1 confocal microscope was used to image samples.

### Immunohistochemistry

Femurs of mice were immersed in 4% paraformaldehyde for 48 h and then decalcified with 0.5 m EDTA (Servicebio) at room temperature. Following continuous shaking for 21 days, samples were dehydrated, paraffin‐embedded, and sectioned 4–5 µm. Osteocalcin (OCN) and tartrate‐resistant acid phosphatase (TRAP) stains were obtained to detect OCN^+^ osteoblast and TRAP^+^ osteoclast within the entire ROI areas. OCN primary antibody (Cat. No gb11233; 1:200) and TRAP staining kit (Cat. No 387A‐1KT) were purchased from Servicebio (Wuhan, China) to measure the number of osteoblasts (N. Ocn^+^) and TRAP‐positive multinucleated osteoclasts (N. Trap+). Three slices with five regions in each slice were analyzed for distal femur with immunohistochemistry. Thus, a total of 15 data points was statistically obtained. Images were acquired with an Olympus optical microscope (Tokyo, Japan). The numbers of osteoblasts or osteoclasts were counted by Image Pro Plus 6.0 software (USA) by cells per bone perimeter (B. Pm).

### RNA Isolation and Quantitative Real‐Time PCR (QPCR)

Total RNA isolation was performed using RNAiso Plus reagent (TaKaRa) and RNA concentration was measured with NanoDrop at OD_260_/OD_280_. The cDNA was obtained by RNA reverse transcription with PrimeScriptTMRT Master Mix (TaKaRa) according to the manufacturer's instructions. QPCR reaction was performed using Green QPCR SuperMix (TransGen Biotech) with a QPCR system (ABI StepOne; Applied Biosystems). Mouse and human *Gapdh* or *β–actin* was used as the internal housekeeping gene, respectively and the expression levels were calculated with 2^−ΔΔCT^ method. The primers of genes used for QPCR were shown in Table S2 (Supporting Information).

### Protein Extraction and Western Blotting

Total proteins were obtained from colon and bone tissues or cells by RIPA (Beyotime Biotechnology) buffer containing with protease inhibitors (Beyotime Biotechnology). BCA protein assay kit (Sangon Biotech) was used to determine the protein concentration. Polyvinylidene fluoride (PVDF) membrane (Millipore) was used to immobilize and block the separated proteins by wet transfer systems (Bio‐Rad). The primary and secondary antibodies and dilution ratio are as below, E‐cadherin (1:1000; Cell Signaling), ZO‐1 (1:1000; Proteintech), AhR (1:2000; Proteintech), β‐catenin (1:5000; Proteintech), β‐Tublin (1:2000; Proteintech), β‐actin (1:5000; Proteintech), GAPDH (1:50 000; Proteintech) and secondary anti‐mouse/rabbit HRP‐conjugated antibodies (SA00001‐1 and SA00001‐2; Proteintech) were subsequently applied. Blots were analyzed by the enhanced‐chemiluminescence (ECL) HRP substrate (Millipore) and ChemiDoc Imager (Bio‐Rad).

### Enzyme‐Linked Immunosorbent Assay (ELISA)

Quantitative measurements of serum CTX, PINP, and IL‐10 were performed according to the manufacturer's instructions (Shanghai Huyu Biotechnology Co., Ltd).

### Statistical Data Analysis

Statistical data analyses were carried out in GraphPad Prism (version 8.0). Correlation network analyses were performed using R software (version 4.2.1) and illustrated with Cytoscape (version 3.7.0). Data were presented as mean ± SD. Unpaired and two‐tailed Student's *t*‐test were used for comparisons between two groups. One‐way ANOVA followed by Tukey's post hoc test was performed among multiple‐groups. For all experiments, *P* < 0.05 was set as the statistical significance.

## Acknowledegments

C.C. and Z.C. contributed equally to this work. This work was financially supported by the Strategic Priority Research Program of the Chinese Academy of Sciences (Grant No. XDB0540300), Hubei Provincial Science and Technology Plan Project (2023BCA005), Natural Science Foundation of Guangdong Province for Distinguished Young Scholars (2022B1515020044) and Foshan Core Technology Tackling Key Project (1920001000262).

## Conflict of Interest

The authors declare no conflict of interest.

## Supporting information

Supporting Information

## Data Availability

The data that support the findings of this study are available from the corresponding author upon reasonable request.
