# Supplementary material for: Microbial Tryptophan Metabolites Ameliorate Ovariectomy‐Induced Bone Loss by Repairing Intestinal AhR‐Mediated Gut‐Bone Signaling Pathway
Source: Adv Sci (Weinh). 2024 Jul 23;11(36):2404545. doi: 10.1002/advs.202404545 (PMC11423200; doi:10.1002/advs.202404545)
Supplement: Supplementary file 1 — Supporting Information [file ADVS-11-2404545-s001.docx]

**Supplemental information**

**Microbial Tryptophan Metabolites Ameliorate Ovariectomy-Induced Bone Loss by Repairing Intestinal AhR-Mediated Gut-Bone Signaling Pathway**

Chuan Chen,^1,2,#^ Zheng Cao,^1,2,#^ Hehua Lei,^1,2^ Cui Zhang,^1,2^ Mengjing Wu,^1,2^ Shaohua Huang,^3^ Xinzhi Li,^4^ Denghui Xie,^*,5^ Maili Liu, ^1,2^ Limin Zhang,^*,1,2^ Gang Chen,^*,6^

^1^State Key Laboratory of Magnetic Resonance and Imaging, National Centre for Magnetic Resonance in Wuhan, Innovation Academy of Precision Measurement Science and Technology, CAS, Wuhan 430071, China

^2^University of Chinese Academy of Sciences, Beijing 100049, China

^3^Institute of Drug Discovery and Technology, Ningbo University, Ningbo 315211, China.

^4^School of Pharmacy and State Key Laboratory of Quality Research in Chinese Medicine, Macau University of Science and Technology, Macau 999078, China

^5^Department of Joint Surgery, Center for Orthopaedic Surgery, the Third Affiliated Hospital of Southern Medical University, Guangzhou 510515, China

^6^Department of Geriatrics, Hubei Provincial Hospital of Traditional Chinese Medicine (Affiliated Hospital of Hubei University of Chinese Medicine), Wuhan 430060, China

^#^These authors contributed equally to this work.

*Correspondence: xiedenghui221122@smu.edu.cn (D. Xie), wofavo@qq.com (G. Chen), or zhanglm@wipm.ac.cn (L. Zhang)


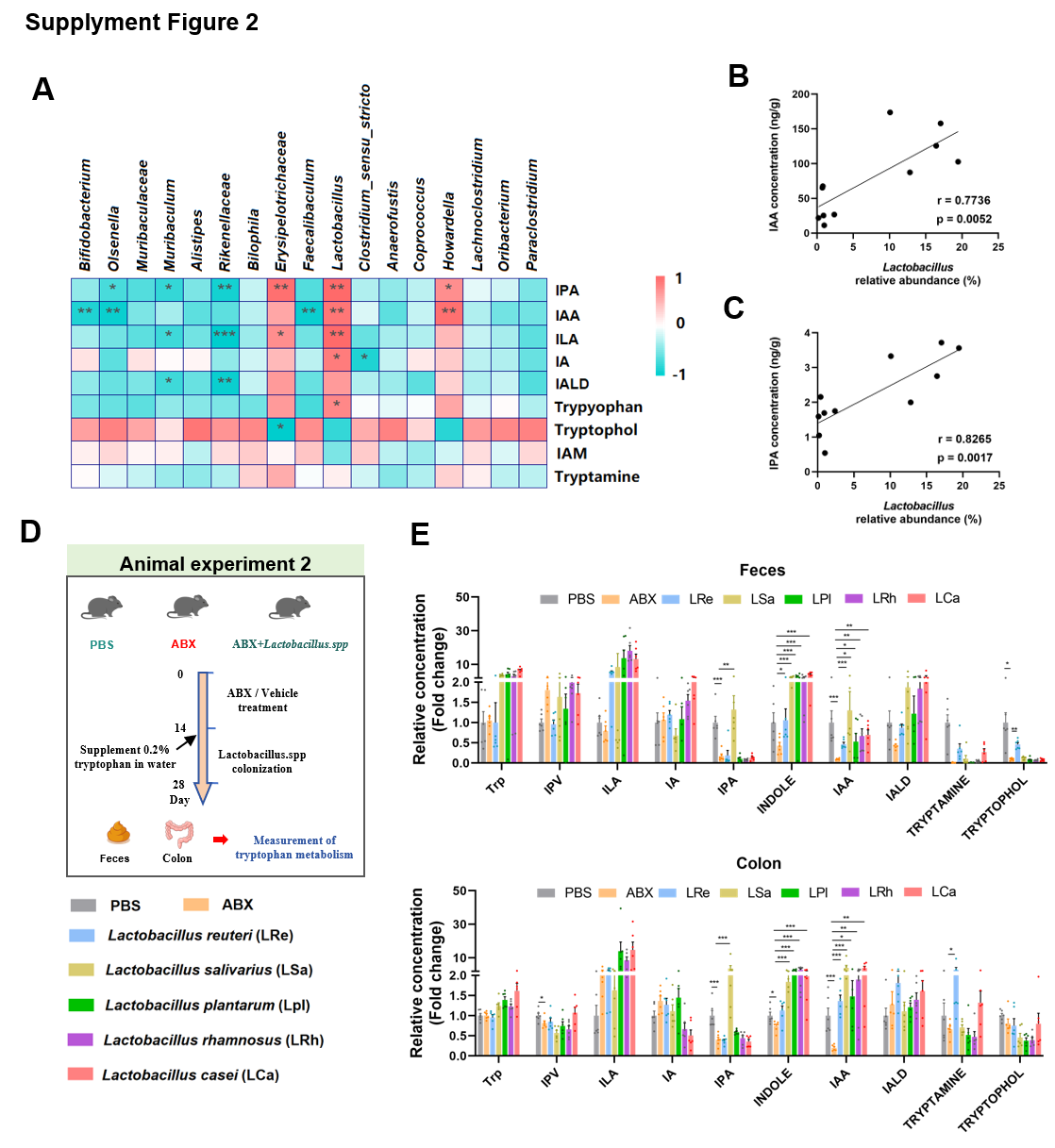


**Figure S1.** A-C) Spearman correlations between the levels of indole metabolites such as IAA and IPA and the gut microbiota, especially *Lactobacillus*. D) Experimental scheme of several *Lactobacillus* species colonization to control mice and antibiotics (ABX)-treated mice. E) Targeted quantification of indole metabolites in feces and colon samples of mice using HPLC-QQQ-MS.

**
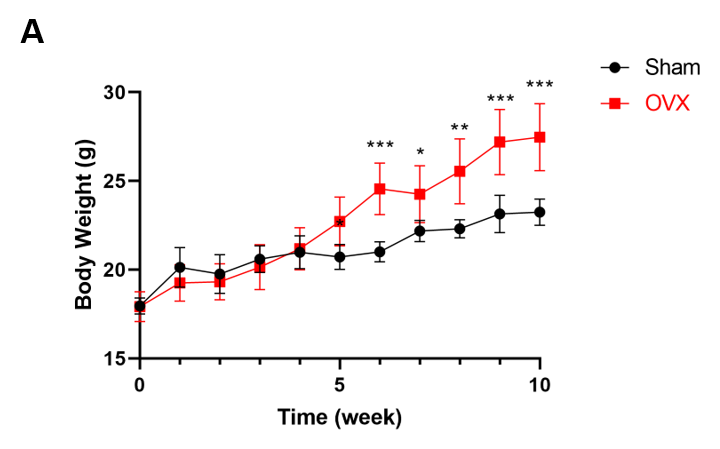
**

**Figure S2.** Body weight changes of Sham and OVX mice. Data are shown as mean ± SD. P values were obtained by two-tailed Student’s t test, *p < 0.05, **p < 0.01, and ***p < 0.001.


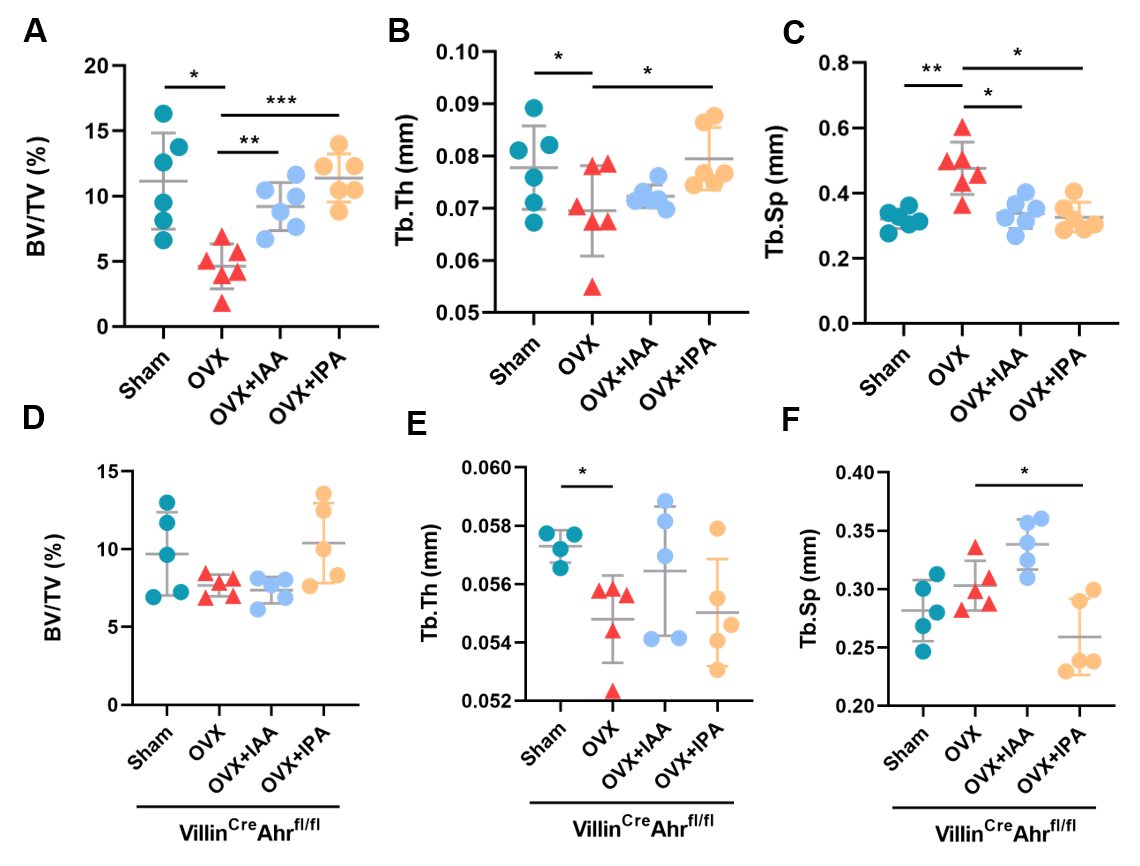


**Figure S3.** The trabecular bone parameters of mice. A-C) Quantitative analysis of 3D parameters for trabecular bone microarchitecture, including BV/TV, Tb.Th, and Tb.Sp in WT mice (n = 6). D-F) Quantitative analysis of 3D parameters for trabecular bone microarchitecture, including BV/TV, Tb.Th, and Tb.Sp in Villin^Cre^Ahr^fl/fl^ mice (n = 5). Data are shown as mean ± SD. P values were obtained by one-way ANOVA with multiple comparisons, *p < 0.05, **p < 0.01, and ***p < 0.001.


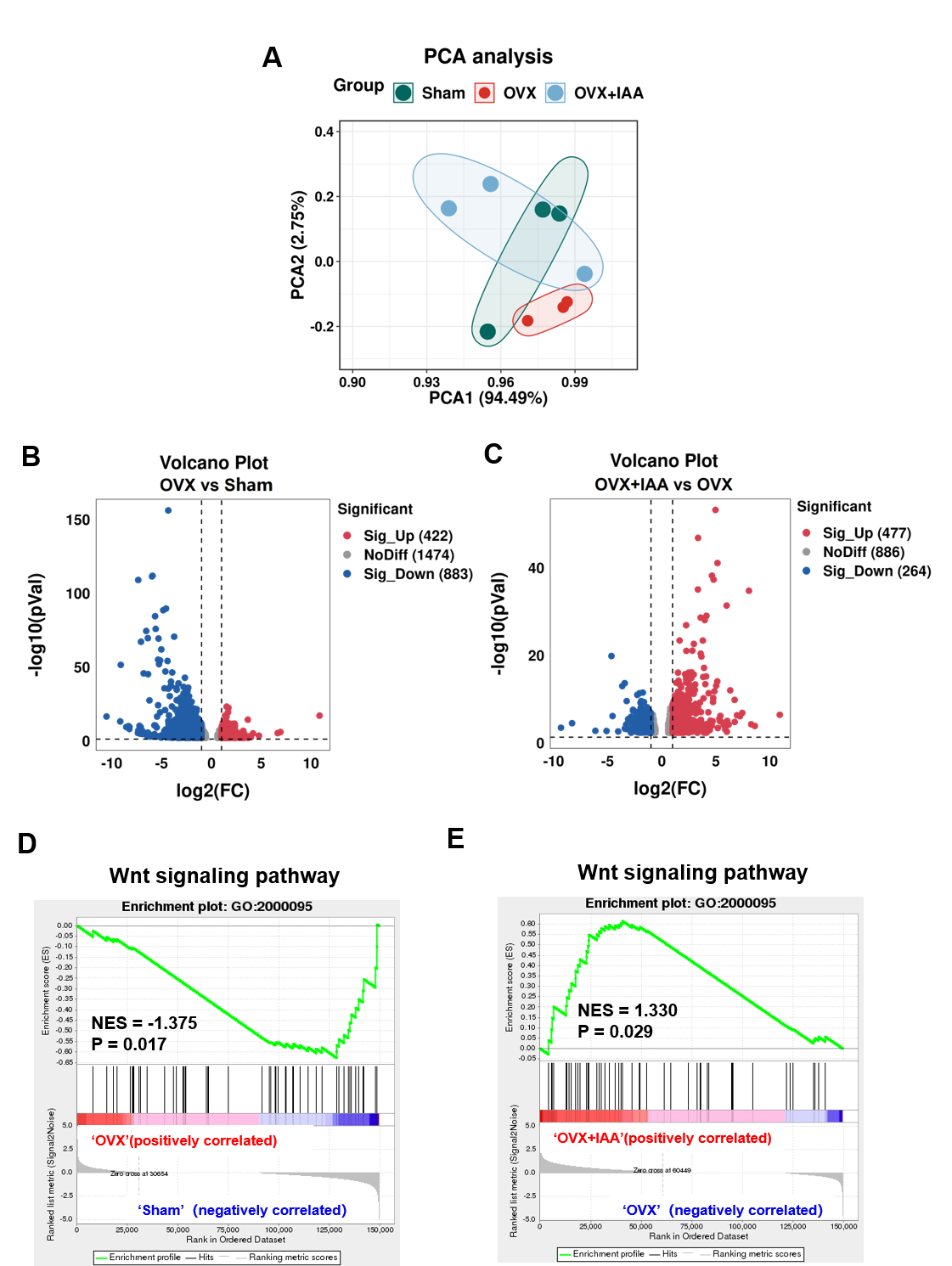


**Figure S4.** Analysis of transcriptome changes in the intestine of OVX, Sham and the IAA-treated mice. A) Principal coordinates analysis (PCA) of transcriptome differences among the Sham (green), OVX (red) and OVX+IAA groups (blue). Ellipses indicate the 95% confidence interval (n = 3). B) Volcano plot showing the changes of colon genes (fold change ≥ 1.5) between OVX and Sham group. C) Volcano plot showing the changes of colon genes (fold change ≥ 1.5) between OVX+IAA and OVX group; D) GSEA revealing the enrichment of differently expressed genes between OVX and Sham group in the Wnt signaling pathway (n = 3). E) GSEA revealing the enrichment of differently expressed genes between OVX+IAA and OVX group in the Wnt signaling pathway (n = 3).


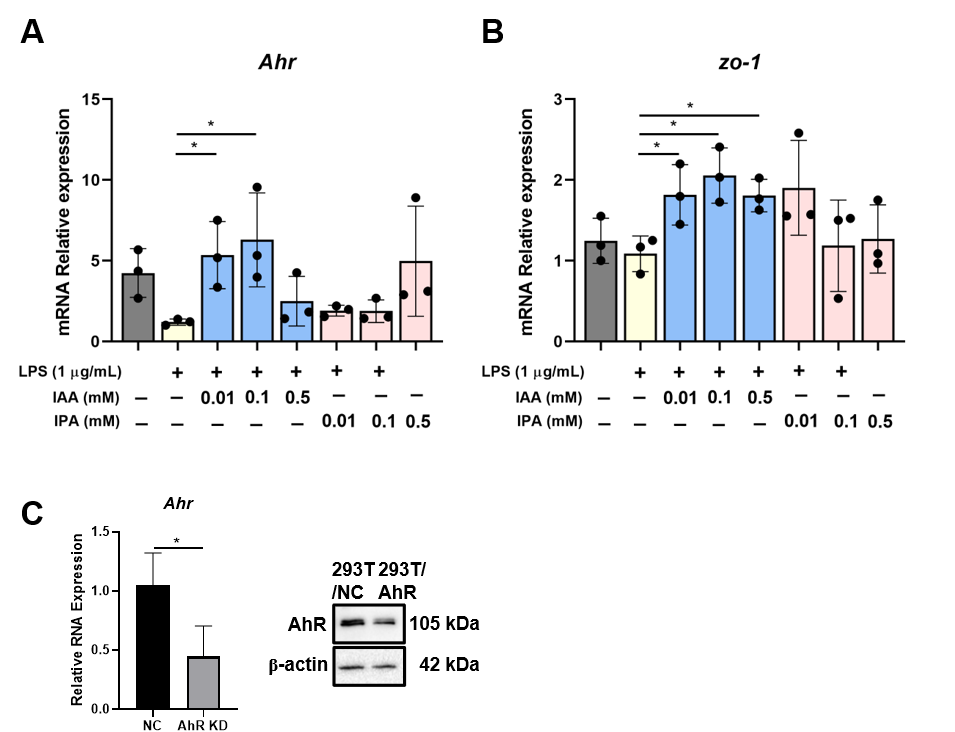


**Figure S5.** Activation of AhR repairs intestinal barrier function. A, B) The mRNA expression of *Ahr* and *Zo-1* in Caco-2 cells with different concentration treatment of IAA and IPA. C) mRNA and protein levels of AhR in 293T knockdown cells.


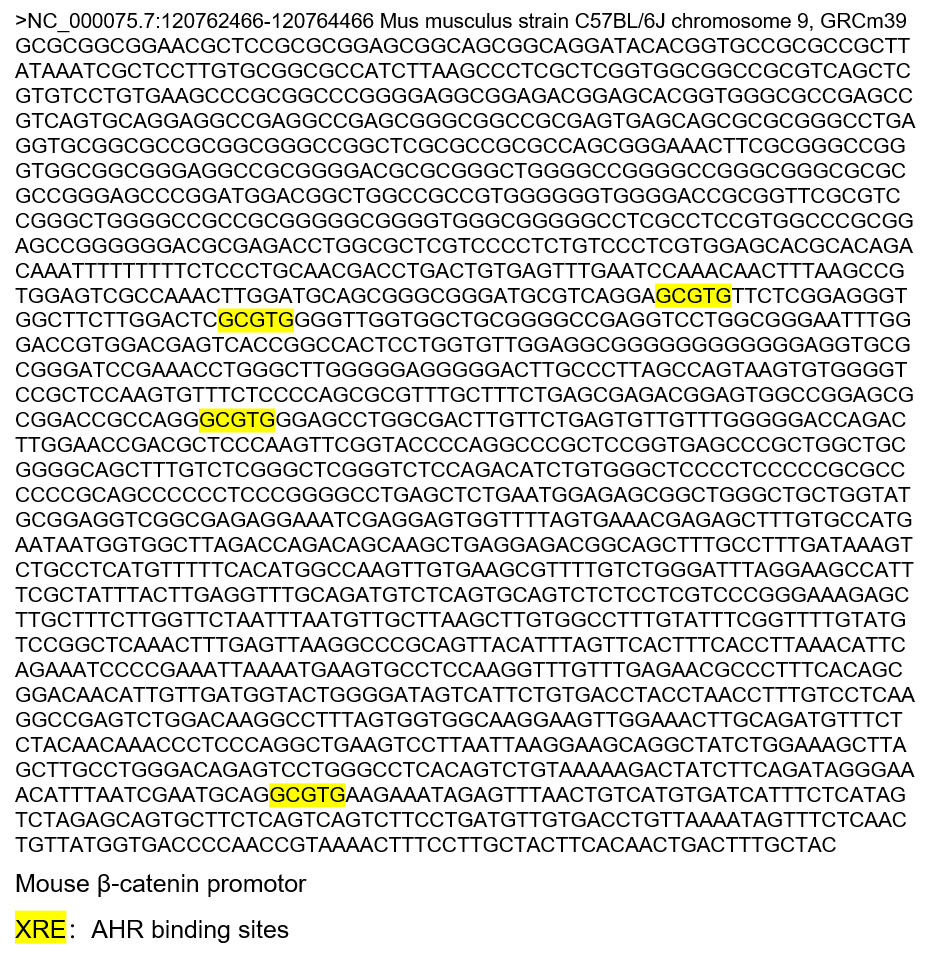


**Figure S6.** Prediction of AHR binding sites in β-catenin gene promotor sequence from -2000 bp to +10 bp.

**Table S1.** Plasmid sequence.

| Reagent or resource | Sequence | |
| --- | --- | --- |
| Plko.1-puro-GFP-NC | | CCGGCCTAAGGTTAAGTCGCCCTCGCTCGAGCGAGGG  CGACTTAACCTTAGGTTTTTG |
| Plko.1-puro-GFP-AHR | | CCGGGCTACCACATCCACTCTAAGCCTCGAGGCTTAGA  GTGGATGTGGTAGCTTTTTG |

**Table S2.** Primers used for quantitative real-time PCR.

|  | Forward primer (5’ -3’) | Reverse primer (5’-3’) |
| --- | --- | --- |
| *Muc2* | GCTGACGAGTGGTTGGTGAATG | GATGAGGTGGCAGACAGGAGAC |
| *Myosin Vb* | CCCCTTCTTTGTAGTCCTTGG | CGTACAGCGAGCTCTACACC |
| *Occludin* | CCTCCAATGGCAAAGTGAAT | CTCCCCACCTGTCGTGTAGT |
| *Claudin* | TTAGCCCTGACCGAGAAAGA | AAAGGACCTCTCTGGTGCTG |
| *Ptprh* | GGTAAAAGTGGGTAGGAAATGGC | GTGGCTGTGTAGGACTGAGC |
| *ZO-1* | GCCAGAGAAAAGTTGGCAAG | TTGGATACCACTGCGCATAA |
| *E-cadherin* | CAGCCTTCTTTTCGGAAGACT | GGTAGACAGCTCCCTATGACTG |
| *Ahr* | CCATGTCCATGTACCAGTGC | GAAAGCCCTTACCTTGCTTAGGA |
| *Alp* | GGACAGGACACACACACAC | CAAACAGGAGAGCCACTTCA |
| *Opg* | ACCCAGAAACTGGTCATCAGC | CTGCAATACACACACTCATCACT |
| *Opn* | AGCAAGAAACTCTTCCAAGCAA | GTGAGATTCGTCAGATTCATCCG |
| *Runx2* | AGGGACTATGGCGTCAAACA | GGCTCACGTCGCTCACTT |
| *Sp7* | ATGGCGTCCTCTCTGCTTG | TGAAAGGTCAGCGTATGGCTT |
| *Col I* | GCTCCTCTTAGGGGCCACT | CCACGTCTCACCATTGGGG |
| *Nfatc1* | CTGTGTTCCCACATGTCCTC | GCGACTGCAGTGTGTTCTTT |
| *Mmp9* | TCCTTGCAATGTGGATGT | CTTCCAGTACCAACCGTCCT |
| *Ctsk M* | GAAGAAGACTCACCAGAAGCAG | TCCAGGTTATGGGCAGAGATT |
| *c-fos* | CGGGTTTCAACGCCGACTA | TTGGCACTAGAGACGGACAGA |
| *Traf6* | TCGGACCCTGGAGGACAA | CCAAACTTGCCAATCTTCCAA |
| *Gapdh* | AAATGGTGAAGGTCGGTGTGAAC | CAACAATCTCCACTTTGCCACTG |
| *Ahr H* | TGGGTCCAGTCTAATGCACG | TGCTCTGTTCCTTCCTCATCT |
| *β-catenin H* | CATCTACACAGTTTGATGCTGCT | GCAGTTTTGTCAGTTCAGGGA |
| *Zo-1 H* | ACCAGTAAGTCGTCCTGATCC | TCGGCCAAATCTTCTCACTCC |
| *β-actin H* | CACCATTGGCAATGAGCGGTTC | AGGTCTTTGCGGATGTCCACGT |
| 338F_806R | ACTCCTACGGGAGGCAGCAG | GGACTACHVGGGTWTCTAAT |
